# Differential expression of cyclin D1 in pituitary neuroendocrine tumours: Relation to aggressive potential

**DOI:** 10.4102/ajlm.v15i1.3077

**Published:** 2026-08-24

**Authors:** Kasiemobi E. Uchime, Nicholas A. Awolola, Charles C. Anunobi, Olufemi B. Bankole, Adekunbiola A. Banjo, Sung-Hye Park

**Affiliations:** 1 Department of Anatomic and Molecular Pathology, Lagos University Teaching Hospital (LUTH), Idi-Araba, Lagos, Nigeria; 2 Department of Anatomic Pathology and Forensic Medicine, Faculty of Basic Clinical Sciences, College of Medicine and Health Sciences, Afe Babalola University Ado-Ekiti (ABUAD) and ABUAD Multi-System Hospital, Ado-Ekiti, Ekiti State, Nigeria; 3 Department of Anatomic and Molecular Pathology, Faculty of Basic Clinical Sciences, College of Medicine, University of Lagos (UNILAG), Lagos, Nigeria; 4 Department of Surgery, Neurosurgery Unit, Lagos University Teaching Hospital (LUTH), Idi-Araba, Lagos, Lagos, Nigeria; 5 Department of Surgery, Neurosurgery Unit, Faculty of Clinical Sciences, College of Medicine, University of Lagos (UNILAG), Lagos, Nigeria; 6 Department of Pathology, College of Medicine, Seoul National University Hospital, Seoul, Republic of Korea

**Keywords:** pituitary neuroendocrine tumours, pituitary adenoma, Cyclin D1, aggressive PitNET, Ki-67, brain invasion

## Abstract

**Background:**

In Nigeria, patients with pituitary neuroendocrine tumours (PitNETs) commonly present late with complications. Early identification of aggressive PitNETs could improve outcomes.

**Objective:**

This study aimed to determine frequency and degree of cyclin D1 expression in PitNETs and compare levels in aggressive versus non-aggressive tumours and normal pituitary.

**Methods:**

Retrospective case–control study (2010–2019) using two tissue microarrays from mitotically active hotspots on paraffin blocks: 70 histologically diagnosed PitNETs and 29 normal pituitary controls. Immunohistochemistry was performed for Ki-67, cyclin D1, anterior pituitary transcription factors, CK MNF116, and hormones. Aggressive PitNETs were classified per 2022 WHO criteria (mitotic rate > 2/10 HPF, Ki-67 ≥ 3% or radiological invasion).

**Results:**

Fourteen (20%) PitNETs were classified as aggressive (8 invasive, 6 proliferative). Median age in the aggressive group was 36.5 years with equal gender distribution. The commonest aggressive subtype was PIT-1 lineage lactotroph PitNET (5/14, 35.7%). Moderate–strong nuclear cyclin D1 expression (IRS ≥ 5) occurred in 85.7% of aggressive PitNETs versus 67.9% of non-aggressive tumours and 0% of controls (*p* < 0.01). Positive predictive value of cyclin D1 for aggressiveness among PitNETs was 24% (12/50 positive cases).

**Conclusion:**

Cyclin D1 is significantly overexpressed in aggressive PitNETs compared with non-aggressive tumours and normal pituitary, suggesting its potential as a biomarker for early detection, risk stratification, and therapeutic targeting, especially in resource-limited settings.

**What the study adds:**

This study demonstrates marked cyclin D1 overexpression in aggressive PitNETs, supporting its use as a biomarker for early detection, more precise risk stratification, and as a potential therapeutic target in resource-limited environments.

## Introduction

Pituitary adenomas, now termed pituitary neuroendocrine tumours (PitNETs) per the World Health Organization (WHO) Classification of Tumors of Endocrine Organs 5th edition (2022),^1^ rank third globally among primary intracranial tumours after meningiomas and gliomas.^2^ In Nigeria, local studies report PitNETs as the second most prevalent intracranial tumour.^3,4,5^ These neoplasms arise from anterior pituitary cells and, though typically benign, may exhibit aggressive behaviour by means of compression or invasion of the optic chiasm or cavernous sinus, causing visual loss and neurological deficits.^6,7^ Despite their clinical significance, pituitary adenomas remain under-investigated in Africa, particularly concerning their immunohistochemical profiles and clinical correlations.

The 2016 European Pituitary Pathology Group,^8^ WHO 2017 (4th edn)^6^ and 2022 (5th edn)^1^ classifications emphasise proliferation markers (mitoses > 2/10 high-power field [hpf], Ki-67 ≥ 3%) with or without invasion (defined by pre-operative magnetic resonance imaging [MRI]/endoscopic intraoperative examination) for the identification of PitNETs with aggressive potential (high-risk PitNETs), discouraging ‘atypical adenoma’ terminology. The 2022 WHO classification of PitNETs uses immunohistochemistry for PIT1, TPIT, and SF1 transcription factors to define lineages: PIT1 (somatotroph/lactotroph/thyrotroph), TPIT (corticotroph), and SF1 (gonadotroph), plus anterior pituitary hormones and other markers.^1,7,8,9^ This reduces null-cell cases by better identifying gonadotroph tumours.^1,7,8,9^ This classification is often integrated with the PitNET’s morphology (e.g. acidophilic densely granulated somatotroph with perinuclear low molecular weight kininogen; chromophobic sparsely granulated with fibrous bodies; basophilic densely granulated corticotroph with diffuse low molecular weight kininogen; Crooke cell with ring-like low molecular weight kininogen).^1,7,8,9^ High-risk or aggressive PitNETs, including those formerly termed atypical adenomas or carcinomas (now metastatic PitNETs), have criteria for identification that are still evolving.^1,7^ Some of the high-risk histotypes include densely granulated PIT-1+ lactotroph, male lactotrophs, immature PIT1-lineage PitNETs, Crooke cell PitNETs, sparsely granulated somatotrophs, and silent corticotrophs.^1,10^ They tend to be non-functioning macroadenomas that exhibit rapid growth, frequently recur after surgical resection and show poor response to conventional therapies.^6,7^ Aside from evidence of invasion, proliferative indices – mitotic count > 2/10 hpf, Ki-67 index ≥ 3% (or ≥ 10% for heightened risk), and extensive p53 immunoreactivity (> 10% nuclei or TP53 mutation) – are indicated for identifying proliferative or aggressive PitNETs per the French five-tiered system (grades 1b–3), aiding prognostication beyond WHO morphology alone.^6,7,8,9,10^ The WHO 2022 classification eliminated ‘atypical adenoma’ terminology (which required all three proliferative markers) because of poor aggressive behaviour prediction.^1,8,9^ The French grading criteria system defines ‘proliferative’ PitNETs (grades 1b/2b) as two or more of the three markers exceeding thresholds: Ki-67 ≥ 3%, mitosis > 2/10 hpf, or p53 > 10% positive nuclei.^9,10^ Immunoreactivity for p53 is not yet validated.^8^ Its use is optional, but can strengthen classification when borderline.^9^

Cyclin D1 dysregulation has been implicated in pituitary tumorigenesis and is associated with increased proliferative activity in PitNETs/adenomas. Consequently, CDK4/6 inhibitors, including palbociclib, have emerged as promising therapeutic agents for aggressive pituitary tumors.^11,12,13^ Although therapeutic agents targeting cyclin D1 are being developed for several cancers, little is known about its expression in PitNETs, particularly in African populations, and its potential role as a biomarker for tumour aggressiveness.

This study aims to assess the cyclin D1 expression pattern in aggressive PitNETs compared to non-aggressive PitNETs and normal anterior pituitary tissues. By investigating cyclin D1 as a possible marker of aggressive clinical behaviour, this research seeks to fill gaps in understanding the molecular pathology of PitNETs in Nigeria and contribute to improved diagnostic and therapeutic strategies for these tumours.

## Methods

### Ethical considerations

Approval for this study was obtained from the Health Research and Ethics Committee of Lagos University Teaching Hospital on 10 June 2019, with the assigned number ADM/DCST/HREC/APP/2830, and adhered to confidentiality and ethical standards during data collection and tissue handling. The research did not involve any physical contact with live patients. Patients’ confidentiality was protected by not disclosing their names and by encrypting their hospital and laboratory numbers. Written informed consent for the retrieval of the normal pituitary tissues during autopsies, included in the study, was obtained from the next-of-kin of the deceased.

### Study design

This 10-year retrospective case-control study was conducted using tissue microarrays constructed from 70 histologically diagnosed PitNETs diagnosed between 01 January 2010 and 31 December 2019, and 29 age-matched and sex-matched normal anterior pituitary samples. The formalin-fixed paraffin-embedded (FFPE) tissue blocks of the 70 histologically diagnosed PitNETs were collected from the archives of the Department of Anatomic and Molecular Pathology, Lagos University Teaching Hospital, Lagos, Nigeria, during the period 10 June 2019 to 10 June 2020. The 29 normal anterior pituitary tissues were collected from autopsies conducted at the Department of Anatomic and Molecular Pathology, Lagos University Teaching Hospital, Lagos, Nigeria, from 10 June 2019 to 10 June 2020.

### Study population

The study population comprised patients diagnosed with pituitary adenomas, identified in histopathology diagnostic records at Lagos University Teaching Hospital. Inclusion criteria consisted of histologically confirmed diagnosis of PitNETs with available FFPE tissue blocks, and corresponding clinical and radiologic data. Normal anterior pituitary tissues, for controls, were obtained from autopsies, with age and sex matched to cases. The normal tissues were processed and confirmed as normal on Haematoxylin and Eosin (H&E) and reticulin stains. Exclusion criteria included inadequate tissue preservation or missing clinical data.

### Materials and tissue processing

Formalin-fixed paraffin-embedded tissue blocks of all histologically diagnosed PitNETs cases were retrieved from the pathology archives, while the controls – normal anterior pituitary tissues collected during autopsies – were fixed with 10% neutral buffered formalin, processed using a Leica TP1020 tissue processor (Leica Biosystems, Nussloch, Germany), and embedded in liquid paraffin to form FFPE tissue blocks. These FFPE tissue blocks of the histologically diagnosed PitNETs and the normal anterior pituitary tissues were collected within the period 10 June 2019 to 10 June 2020. Sections (4 µm – 5 µm) were cut from all the FFPE blocks of the PitNETs and normal anterior pituitary tissues for histological examination. Clinical information of the patients diagnosed with PitNETs included in the study was obtained from the medical records of Lagos University Teaching Hospital. Pre-operative radiological reports (brain MRI or computed tomography) and operative findings were retrieved to assess tumour invasiveness. Invasive tumors were recognised radiologically, as those in Knosp grades 3 to 4 (cavernous sinus invasion) or Hardy C-E (sellar or sphenoid invasion) as recommended in the WHO 2017 (4th edn) Classification of Tumors of Endocrine Organs.^6,14,15^

### Histopathological review

Haematoxylin and Eosin and reticulin stains were performed on the sectioned FFPE tissue blocks to confirm tumour presence and normal tissue status. All slides were independently reviewed by two experienced pathologists to confirm diagnoses and identify the regions of interest. Mitotic activity on the H&E-stained slides was quantified both manually and automatically, by counting abnormal mitoses per 10 hpf using Sectra software (Sectra AB, Teknikringen 20, SE-583 30 Linkoping, Sweden). Hotspot areas, exhibiting the highest mitotic activity, were identified on H&E-stained slides and circled for further analysis. Two tissue microarrays were constructed from circular punches of 2 mm diameter from these hotspots and randomly from non-diseased tissues (a maximum of 60 cores per tissue microarray block).

### Immunohistochemistry

Immunostaining was performed on tissue sections from tissue microarrays for PitNET typing and proliferation markers. Primary antibodies utilised included anterior pituitary transcription factors (TPIT, PIT-1, & SF-1), anterior pituitary hormones (ACTH, GH, LH, FSH, TSH, PRL), CK MNF116, cyclin D1, and Ki-67, with protocols adapted from standard manufacturer guidelines and performed on the Ventana Benchmark ULTRA system (Roche Diagnostics, Basel, Switzerland). The summary of the immunohistochemistry protocols of the antibodies used in the study is (for details see Online Supplementary Table 1):

- Cyclin D1 (SP4 rabbit monoclonal, Ventana, ready-to-use, CC1 64 min).
- Ki-67 (MIB-1, Dako,1:100, CC 132 min).
- Transcription factors: TPIT (Sigma AMab 91409, 1:200), PIT-1 (Invitrogen Thermo PA5-59662, 1:100), SF-1 (Abcam Ab217317, 1:100).
- Hormones: ACTH (Dako A0571, 1:500), GH (Dako A0570, 1:4000), PRL (Ventana EP193, 1:200), LH/FSH/TSH (Dako, per protocol).
- CK (MNF116, Dako M0821, 1:300).

**TABLE 1:** Age and gender distribution of the aggressive and non-aggressive pituitary neuroendocrine tumours analysed in the study, Department of Anatomic and Molecular Pathology, Lagos University Teaching Hospital, Lagos, Nigeria, 10 June 2019 – 10 June 2020.

Gender	*n*	%	Age						Interquartile range	Q1	Q3
			Mean	s.d.	Median	Mode	Minimum	Maximum			
**Aggressive PitNETs**											
Male	7	50.0	35.00	17.17	39.0	NIL	15	59	19.0	23.5	42.5
Female	7	50.0	38.71	16.99	34.0	NIL	23	58	23.3	25.8	49.0
Total	14	100.0	36.86	14.77	36.5	NIL	15	59	17.7	25.8	43.5
**Non-aggressive PitNETs**											
Male	36	64.3	45.00	13.76	47	47	15	69	17.5	36.8	54.3
Female	20	35.7	45.50	14.41	45	43	18	68	16.5	40.5	57.0
Total	56	100.0	45.20	13.86	47	43	15	69	18.5	36.8	55.3
**Total PitNETs**											
Male	43	61.0	43.65	14.9	47	47	15	69	18.0	35.0	53.0
Female	27	39.0	43.25	13.0	43	43	18	68	24.5	32.0	56.5
Total	70	100.0	43.51	14.2	45	43	15	69	20.75	34.0	54.75

s.d., standard deviation; PitNETs, pituitary neuroendocrine tumours; Q1, first quartile; Q3, third quartile.

Positive controls used included tonsil tissue for Ki-67 and cyclin D1. The staining was visualised with appropriate immunostaining detection kits (3,3’-diaminodbenzidine detection with haematoxylin counterstain), and interpretation of nuclear staining was conducted by two independent pathologists, with positive staining indicated by brown nuclei and negative by blue nuclei. Cytoplasmic staining was disregarded. The Ki-67 labeling index was quantified via digital image analysis, and cyclin D1 expression was scored using the immunoreactive score (IRS) method.

### Scoring and grouping

The Ki-67 labeling indices were calculated automatically using digital image analysis on the digital image of the immunoslide using an LG L22WTQ-SF Flatron monitor (LG Electronics, Seoul, South Korea) and the nuclear V9 algorithm (Aperio ScanScope XT System; Aperio Technologies, Inc., Vista, California, United States). The findings were then confirmed manually by virtually evaluating 1000 cells counted from tissue microarray hot spot cores with the aid of a reticle at x400 magnification. The number of Ki-67 positive tumour cells was divided by the total number of cells counted and multiplied by 100 to determine the Ki-67 labeling index (% positive). Cyclin D1 expression was semi-quantitatively scored based on staining intensity (0–3) and percentage of positive nuclei (0–4). The IRS was calculated by multiplying these scores, with resultant scores categorised as negative (IRS score 0), weak (IRS score 1–4), moderate (IRS score 5–8), or strong (IRS score 9–12) expression. Tumours exhibiting high proliferative index (mitotic rate > 2/10 hpf and Ki-67 index ≥ 3%), or evidence of invasion on imaging were classified as aggressive, following WHO criteria (WHO 5th edn).^1^

### Statistical analysis

Data were entered and analysed using SPSS Statistical software for Windows, version 25.0 (IBM Corp., Armonk, New York, United States). Proportions of various tumour types and aggressive phenotypes were compared across groups. Fisher’s exact test was used to evaluate differences in cyclin D1 expression among normal pituitary tissues, non-aggressive, and aggressive PitNETs, with significance set at *p* < 0.05. Tests of association between variables were assessed using Gamma (> 0.7 = strong), Cramer’s V (> 0.5 = moderate), and Kendall’s tau-b (> 0.6 = strong) with significance set at *p* < 0.05. Positive predictive value (PPV) was calculated for cyclin D1 (moderate and strong nuclear expression; IRS ≥ 5), predicting aggressiveness. Results were presented as tables and bar charts, with photomicrographs illustrating representative cases.

## Results

The study included PitNETs with ages 15–69 years (median 45; interquartile range [IQR] 34.0–54.8 years). Aggressive PitNETs (*n* = 14) had a median age of 36.5 years (IQR 25.8–43.5), and a male-to-female ratio of 1:1. The median ages of aggressive PitNETs were 39 years (IQR 23.5–42.5) in male patients and 34 years (IQR 25.8–49.0) in female patients (Table 1).

**FIGURE 1:**
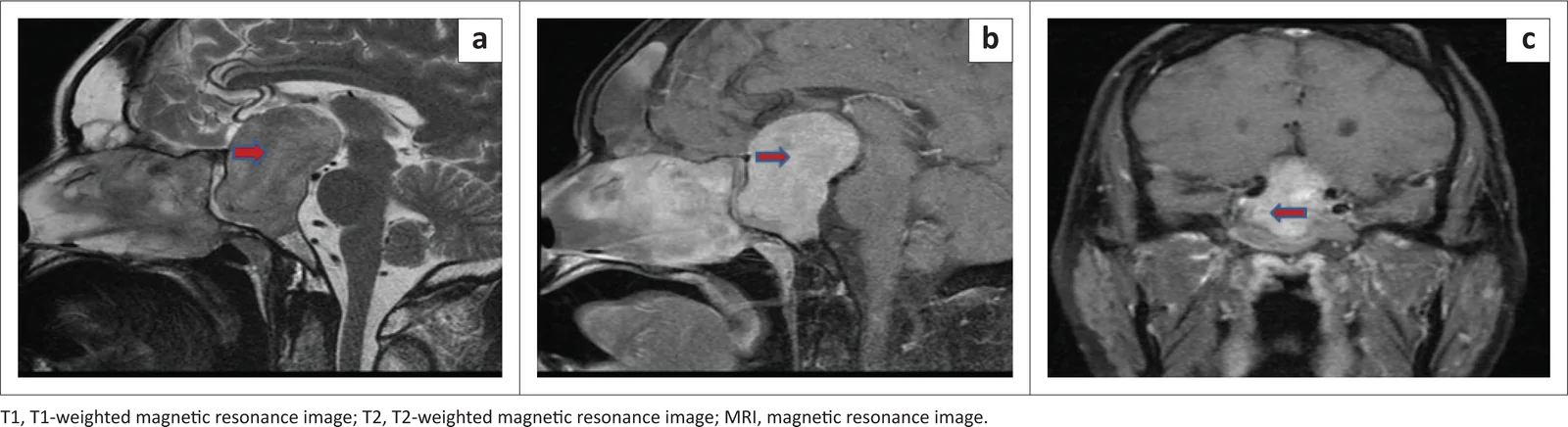
Brain magnetic resonance images of one of the invasive pituitary neuroendocrine tumours (giant adenoma) seen in the study, Department of Anatomic and Molecular Pathology, Lagos University Teaching Hospital, Lagos, Nigeria, 10 June 2019 – 10 June 2020: (a) Sagittal T2-weighted brain MRI without contrast showing a giant suprasellar mass (arrow) with hypointense marrow changes in the clivus suggesting infiltration (Hardy’s grade III); (b) Sagittal T1-weighted brain MRI with contrast showing a hyperintense sellar mass (arrow) with suprasellar extension, the mass has eroded the floor of the sella turcica as well as the upper clivus and it compresses the optic chiasma displacing it superiorly (Hardy’s grade III); (c) Coronal T1-weighted brain MRI with contrast showing an invasive suprasellar mass (arrow) with infiltration of the cavemous sinus as well as partial encasement of the intemal carotid arteries on both sides (Knosp’s grade III).

Imaging using MRI and computed tomography identified eight invasive PitNETs, with representative MRI images of one case shown in Figure 1. No surgically or microscopically invasive PitNETs or pituitary carcinomas were identified.

**FIGURE 2:**
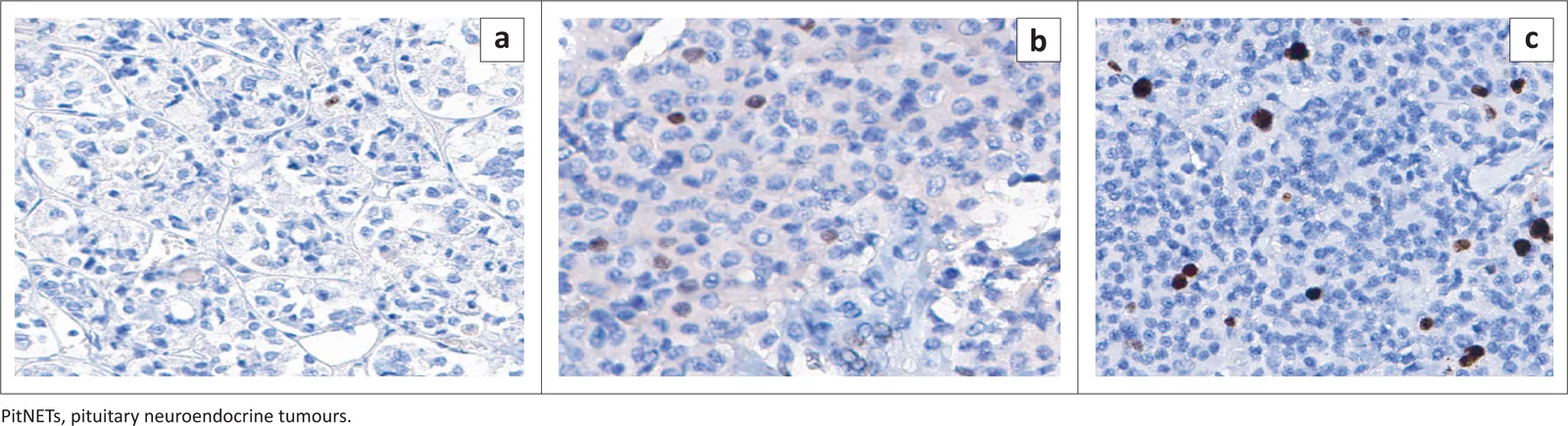
Photomicrographs showing Ki-67 expression pattern in one of the normal anterior pituitary tissues, non-aggressive and aggressive pituitary neuroendocrine tumours seen in the study, Department of Anatomic and Molecular Pathology, Lagos University Teaching Hospital, Lagos, Nigeria, 10 June 2019 – 10 June 2020: (a) Photomicrograph showing negative Ki-67 nuclear expression in one of the normal anterior pituitary tissues seen in the study (400x magnification); (b) Photomicrograph showing Ki-67 expression (Ki-67 labeling index ≤ 3%) in one of the non-aggressive PitNETs with nuclear staining of the proliferating cells (400x magnification); (c) Photomicrograph showing Ki-67 expression (Ki-67 labeling index > 3%) in one of the aggressive PitNETs seen in the study with nuclear staining of the proliferating cells (400x magnification).

Mitotic counts ranged from 1 to 15, and Ki-67 labeling indices ranged from 0.0% to 9.32% across all PitNETs (Table 2). Using the WHO criteria, eight PitNETs were invasive, and six were proliferative (mitotic activity > 2/10 hpf and Ki-67 index ≥ 3%), totaling fourteen aggressive PitNETs. None was both invasive and proliferative. The remaining 56 adenomas were classified as non-aggressive. Lactotroph PitNETs from PIT-1 lineage (*n* = 5) were the most frequent aggressive subtypes, accounting for 35.7% of aggressive adenomas (Table 3). Among the six proliferative aggressive PitNETs, the majority (*n* = 4) were of PIT-1 lineage (three PIT-1 lineage lactotroph PitNETs – two densely granulated and one sparsely granulated; all male patients, and 1 PIT-1 lineage somatotroph PitNET). The remaining proliferative PitNETs lineages were null cell (*n* = 1) and plurihormonal (*n* = 1) PitNETs. However, among the eight invasive aggressive PitNETs, half (*n* = 4) were of the SF-1 lineage. The remaining invasive PitNETs included two null-cell PitNETs, one plurihormonal PitNET, and one PIT-1 lineage lactotroph PitNET. Ten aggressive PitNETs were macroadenomas (1 cm – 4 cm), and four were giant adenomas (> 4 cm). All but one aggressive PitNETs were functional tumours. Examples of Ki-67 immunostaining patterns in normal pituitary, non-aggressive, and aggressive PitNETs are presented in Figure 2.

**TABLE 2a:** Cyclin D1 expression patterns and proliferative features of each group of pituitary tissues seen in the study, Department of Anatomic and Molecular Pathology, Lagos University Teaching Hospital, Lagos, Nigeria, 10 June 2019 – 10 June 2020.

Cyclin D1 expression pattern		Negative		Weak		Moderate		Strong		Total	
		*n*	%	*n*	%	*n*	%	*n*	%	*n*	%
Pituitary tissue group	Normal pituitary tissue	22	75.9	7	24.1	0	0.0	0	0.0	29	100.0
	Non-aggressive PitNETs	5	8.9	13	23.2	8	14.3	30	53.6	56	100.0
	Aggressive PitNETs	1	7.1	1	7.1	2	14.3	10	71.4	14	100.0
Cycin D1 expression in aggressive PitNETS	Invasive PitNETs	0	0.0	1	12.5	2	25.0	5	62.5	8	100.0
	Proliferative PitNETs	1	16.7	0	0.0	0	0.0	5	83.3	6	100.0

**TABLE 2b:** Cyclin D1 expression patterns and proliferative features of each group of pituitary tissues diagnosed at the Department of Anatomic and Molecular Pathology, Lagos University Teaching Hospital, Nigeria between January 1, 2010 and December 31, 2019.

Proliferative features of each group of pituitary tissues		Ki-67 labeling index range (%)	Mitotic count range	Total number of tissues
Pituitary tissue group	Normal pituitary tissue	0.00% – 0.10%	0–1/10 hpf	29
	Non-aggressive PitNETs	0.00% – 2.75%	0–15/10 hpf	56
	**Aggressive PitNETs**			
	Invasive	0.10–2.19%	1–6/10 hpf	8
	Proliferative	3.08–9.32%	3–15/10 hpf	6

**TABLE 2c:** Cyclin D1 expression patterns and proliferative features of each group of pituitary tissues seen in the study, Department of Anatomic and Molecular Pathology, Lagos University Teaching Hospital, Lagos, Nigeria, 10 June 2019 – 10 June 2020.

Total PitNETs cyclin D1 expression pattern	Ki-67 labeling index group									
	0% – 1%		> 1% – 2%		> 2% – < 3%		≥ 3%		Total	
	*n*	%	*n*	%	*n*	%	*n*	%	*n*	%
Negative	2	33.33	3	50.00	0	0.00	1	16.67	6	100.00
Mild	13	92.86	0	0.00	1	7.14	0	0.00	14	100.00
Moderate	9	90.00	0	0.00	1	10.00	0	0.00	10	100.00
Strong	26	65.00	5	12.50	4	10.00	5	12.50	40	100.00

**Total**	**50**	**71.43**	**8**	**11.43**	**6**	**8.57**	**6**	**8.57**	**70**	**100.00**

PitNETs, pituitary neuroendocrine tumours.

Fisher’s Exact test cross tabulation of cyclin D1 expression patterns and the pituitary groups showed a *p*-value of < 0.001, i.e. statistically significant; null hypothesis is rejected.

Proliferative adenomas were recognised using both mitotic rate > 2/10 hpf and Ki-67 labeling index > 3%.

Fisher’s Exact test cross tabulation of cyclin D1 expression patterns and the Ki-67 labeling index group showed a *p*-value of 0.071, i.e. statistically not significant.

**TABLE 3:** Hormonal immunophenotypes of the aggressive and non-aggressive pituitary neuroendocrine tumours analysed in the study, Department of Anatomic and Molecular Pathology, Lagos University Teaching Hospital, Lagos, Nigeria, 10 June 2019 – 10 June 2020.

PitNET transcription factor lineage	PitNET subtype	Immunophenotype	Non-aggressive PitNETs group		Aggressive PitNETs group		Total	
			*n*	%	*n*	%	*n*	%
Null-cell	Null cell PitNET	No markers	14	25.0	2	14.3	16	22.9
TPIT	Corticotroph PitNET	ACTH	2	3.6	0	0.0	2	2.9
PIT1	Thyrotroph PitNET	TSH-beta, alpha subunit	1	1.7	0	0.0	1	1.4
	Somatotroph PitNET	GH ± PRL	3	5.4	1	7.1	4	5.7
	Lactotroph PitNET	PRL, PRL + GH (focal or inconstant)	7	12.5	5	35.7	12	17.1
SFT-1	Gonadotroph PitNET	FSH-beta	27	48.2	4	28.6	31	44.3
Plurihormonal	Plurihormonal PitNET	GH + PRL + ACTH (*N* = 3)	2	3.6	2	14.3	4	5.7
		PRL + TSH + FSH (*N* = 1)	-	-	-	-	-	-

**Total**	**-**	**-**	**56**	**100.0**	**14**	**100.0**	**70**	**100.0**

PitNETs, pituitary neuroendocrine tumours; ACTH, adrenocorticotropic hormone; TSH, thyrod-stimulating hormone; GH, growth hormone; PRL, prolactin; FSH, follicle-stimulating hormone; LH, luteinising hormone.

There was no double adenoma or LH-gonadotroph PitNET immunophenotype seen in this study.

**FIGURE 3:**
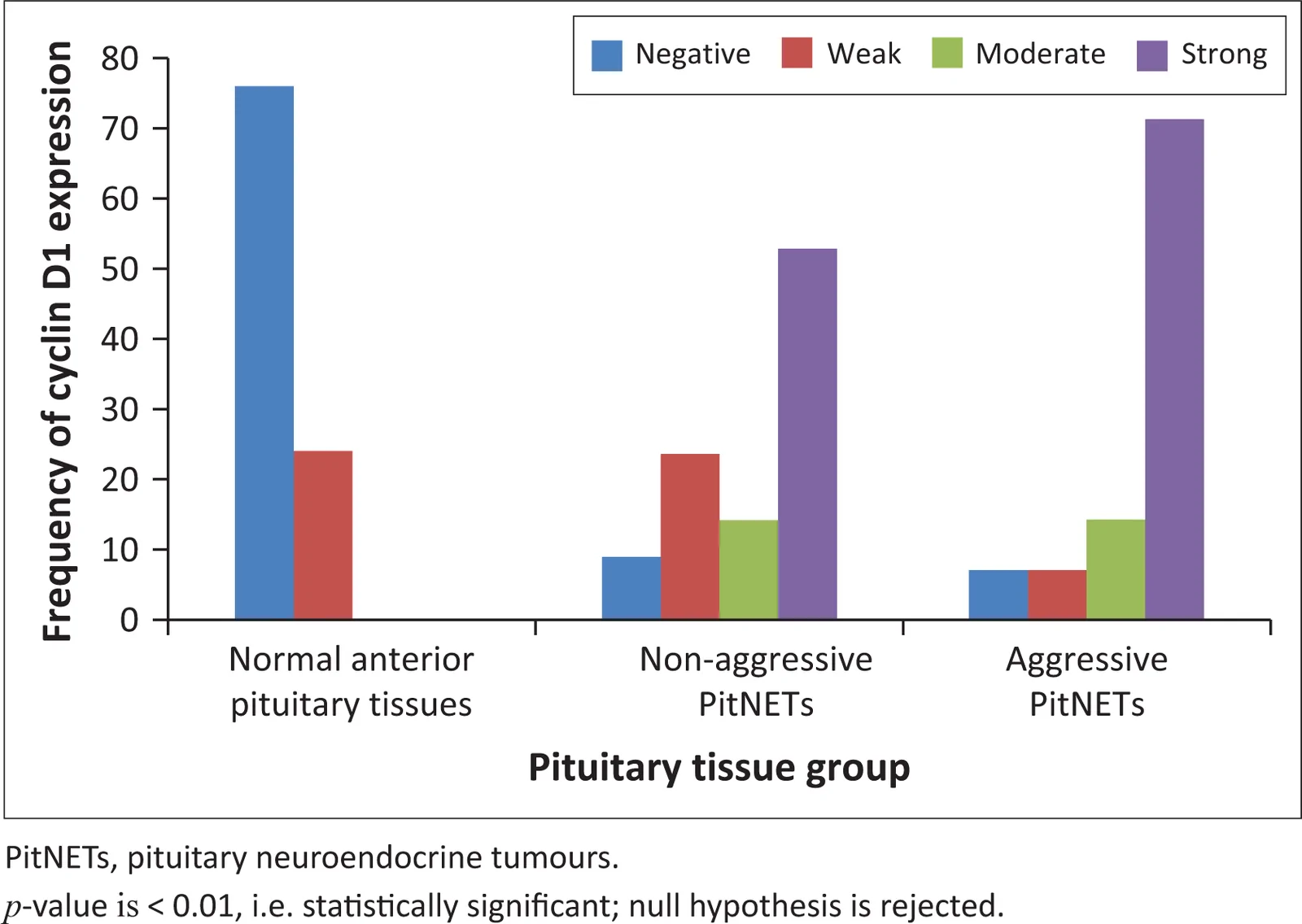
Bar chart showing cyclin D1 expression pattern in normal anterior pituitary tissues, non-aggressive pituitary neuroendocrine tumours and aggressive pituitary neuroendocrine tumours seen in the study, Department of Anatomic and Molecular Pathology, Lagos University Teaching Hospital, Lagos, Nigeria, 10 June 2019 – 10 June 2020.

Fisher’s test with cross-tabulation between cyclin D1 expression and pituitary groups (aggressive PitNETs, non-aggressive PitNETs and normal anterior pituitary tissues) was performed, with the cyclin D1 expression pattern in each group represented in Table 2. Among 29 normal anterior pituitary tissues, 22 (75.9%) showed no cyclin D1 expression, and 7 (24.1%) showed weak expression; none showed moderate or strong expression (Table 2, Figure 3). Of the 56 non-aggressive PitNETs, 5 (8.9%) had no expression, 13 (23.2%) weak, 8 (14.3%) moderate, and 30 (53.6%) strong cyclin D1 expression. Among 14 aggressive PitNETs, 1 (7.1%) had no expression, 1 (7.1%) weak, 2 (14.3%) moderate, and 10 (71.4%) strong nuclear cyclin D1 expression (Table 2, Figure 3).

Moderate and strong cyclin D1 expression was more prevalent in aggressive PitNETs (85.7%) compared to non-aggressive PitNETs (67.9%). No moderate or strong expression was observed in normal tissues. Weak or no expression was observed in 14.2% of aggressive and 32.1% of non-aggressive PitNETs.

Association tests showed a strong positive correlation between cyclin D1 expression and PitNET aggressiveness (Goodman and Kruskal’s gamma = 0.8302; Cramer’s V = 0.5317; Kendall’s tau-b = 0.6006; *p* < 0.01).

**FIGURE 4:**
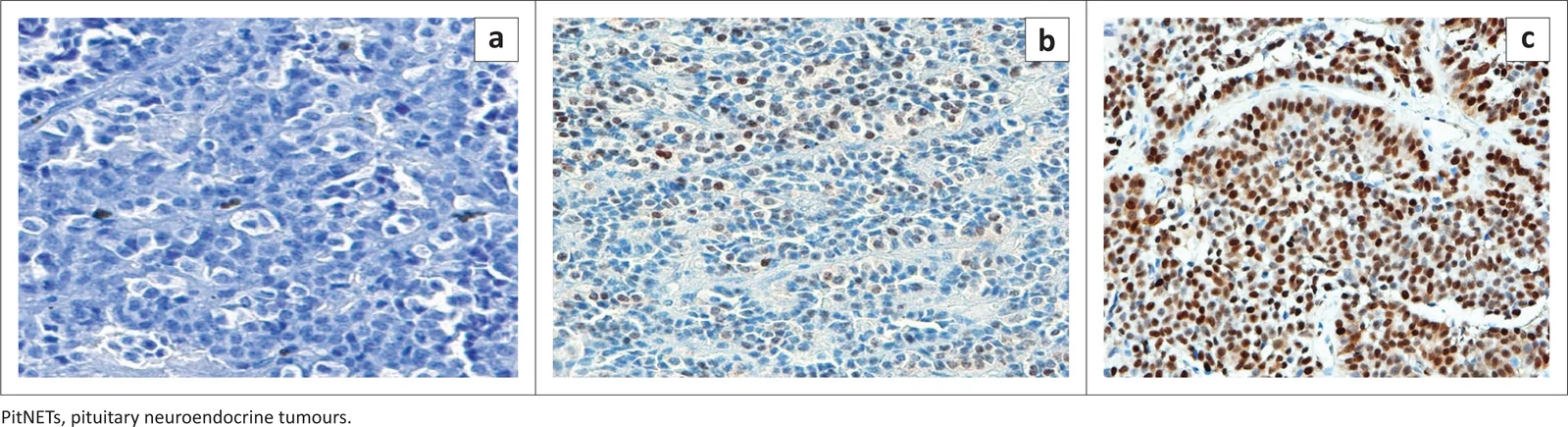
Photomicrographs showing pattern of cyclin D1 nuclear expression in one of the normal anterior pituitary tissues, non-aggressive and aggressive pituitary neuroendocrine tumours as seen in this study conducted at the Department of Anatomic and Molecular Pathology, Lagos University Teaching Hospital, Lagos, Nigeria, from 10 June 2019 to 10 June 2020: (a) Photomicrograph showing negative Cyclin D1 nuclear expression in one of the normal anterior pituitary tissues seen in the study (400x magnification); (b) Photomicrograph showing moderate Cyclin D1 expression, IRS:6 (intensity = 2, percentage = 3) in one of the non-aggressive PitNETs seen in the study (400x magnification); (c) Photomicrograph showing strong nuclear stain for Cyclin D1, IRS: 12 (intensity = 3, percentage = 4) in one of the aggressive PitNETs.

Of the eight invasive PitNETs, five (62.5%) showed strong, two (25.0%) moderate, and one (12.5%) mild cyclin D1 nuclear expression (Table 2). Of the six proliferative PitNETs, five (83.3%) showed strong expression, and one (16.7%) had no expression (Table 2). Cyclin D1 immunostaining of normal pituitary, non-aggressive, and aggressive PitNETs is shown in Figure 4.

Cross-tabulation between cyclin D1 expression with Ki-67 labeling index categories (0% – 1%, > 1% – 2%, > 2% – < 3%, ≥ 3%) and association tests between the two variables showed no statistically significant association (*p* = 0.071; Gamma = 0.1821; Cramer’s V = 0.2873; Kendall’s tau-b = 0.0941) (Table 2).

Moderate/strong cyclin D1 expression (IRS ≥ 5) yielded a PPV of 24% (12 true positives/50 positives) for aggressiveness among PitNETs (*n* = 70), with significantly higher prevalence in aggressive cases (85.7% vs 67.9% in non-aggressive; Fisher’s exact test, *p* < 0.01; Gamma = 0.83).

Based on these results, the null hypothesis that ‘Cyclin D1 expression does not increase in aggressive PitNETs compared to non-aggressive PitNETs and normal tissues’ was rejected. This shows a positive association between moderate to strong cyclin D1 expression in PitNETs with increased aggressive potential. However, the low PPV of 24% suggests that a larger sample size may be required for the assessment of the use of moderate to strong cyclin D1 expression in predicting high-risk or aggressive PitNETs.

## Discussion

This study aimed to characterise the expression pattern of cyclin D1 in PitNETs, including both aggressive and non-aggressive cases, with the goal of evaluating its potential use as a marker to identify aggressive PitNETs for possible future individualised management strategies. About 20% of the PitNETs (14 of 70) were classified as aggressive, comparable to the proportion of pituitary adenomas exhibiting increased proliferative activity reported by Salami et al.,^16^ who identified 21% of the PitNETs in their study as ‘atypical adenomas’ (currently termed high-risk or aggressive PitNETs) defined by Ki-67 ≥ 3%.

Our finding that no cases exhibited both radiologic invasion and proliferative markers reflects the inconsistent relationship between Ki-67 expression and tumour invasiveness reported in the literature. While some studies have demonstrated a significant association between increased Ki-67 proliferation index and radiological tumour invasion, others have found Ki-67 to be non-predictive of tumour invasiveness.^17,18^ However, Salami et al.^16^ reported a higher frequency of microscopic dural invasion (38.6%) with concomitant high Ki-67 expression in a subset of invasive tumours. The radiologically invasive rate of 11.4% observed in our study is comparable to MRI-based series reporting invasion rates of approximately 10%.^19^ However, it is considerably lower than the higher invasion rates reported in studies using intra-operative or histopathological criteria, including the 40% surgically determined (intra-operative) invasion reported by Selman et al.^20^ and the 45.5% microscopic dural invasion reported by Meij et al.^21^ These differences are likely attributable to variations in the definition and assessment of tumour invasion across studies. Whereas our study defined invasion using radiological criteria (MRI-based Knosp grades 3–4 and Hardy grades C–E), other investigators employed direct intra-operative assessment or histopathological examination of resected dura, both of which may identify tumour invasion that is not evident on imaging alone, thereby resulting in higher reported invasion rates.^22^

Most aggressive tumours were PIT-1 lineage prolactinomas, consistent with Liu et al.’s^23^ work linking higher cyclin D1 expression to invasive prolactinomas. The rarity of pituitary carcinomas in our sample aligns with the known low incidence of 0.1% to 0.2% of pituitary carcinomas among pituitary tumours worldwide.^24^

Regarding cyclin D1, nuclear staining being confined to tumour cells matches previous international studies.^6,25,26,27^ Our study demonstrates a sequential increase in cyclin D1 expression from normal pituitary to non-aggressive and then aggressive PitNETs, corroborating findings by Liu et al.,^23^ Jordan et al.,^25^ Turner et al.,^26^ and Hewedi et al.^27^ While normal anterior pituitary tissues mostly lacked strong cyclin D1 expression in our study, weak staining in about 24% of cases is similar to findings of reports by Jordan et al.^25^ Lack of any nuclear expression of cyclin D1 in normal anterior pituitary tissues reported by Hewedi et al.^27^ might be because of their use of smaller normal anterior pituitary tissue sample size.^27^

The higher prevalence of strong or moderate cyclin D1 expression in aggressive PitNETs (85.7%) compared to non-aggressive ones (67.9%) supports a significant association between cyclin D1 expression and tumour aggressiveness, consistent with multiple studies across different populations.^18,23,25,26,27^ These observations suggest that deregulation of cell cycle control, potentially through cyclin D1-related pathways affecting the G1-S phase transition, may contribute to PitNET tumourigenesis. The moderate-to-strong cyclin D1 nuclear expression (IRS ≥ 5) yielded a PPV of 24% for identifying high-risk or aggressive PitNETs in this study, indicating low specificity as a standalone diagnostic predictor since 76% of positive cases were non-aggressive tumours. This modest PPV underscores the need for cyclin D1 to be integrated with established markers such as Ki-67 index and radiological invasion for improved risk stratification, while highlighting its greater value as a sensitive adjunct rather than a definitive diagnostic tool for PitNETs. Although cyclin D1 is not a specific marker, its overexpression could serve as a sensitive indicator for identifying PitNETs with aggressive potential.

### Study limitation and future consideration

A limitation of this study is that tissue microarrays were constructed using only the mitotically active hotspots identified on FFPE tissue blocks. This approach might have inadvertently excluded some immunohistochemically reactive areas, which could affect the representativeness of cyclin D1 and Ki-67 expression assessed. Additionally, a relatively small sample size was used in our study, which may impact on the generalisability of the findings. Future research incorporating molecular genetic analyses and larger cohorts could clarify the mechanisms linking cyclin D1 expression to aggressive phenotypes and potentially identify targets for therapeutic intervention.

### Conclusion

In conclusion, this study reveals that strong cyclin D1 expression is strongly associated with aggressive PitNETs, enhancing our understanding of PitNET biology and encouraging further exploration of the potential use of cyclin D1 for early detection, prognostication, and possible development of novel treatments for aggressive PitNETs. For PitNETs with moderate or strong cyclin D1 expression (IRS ≥ 5), we recommend intensified surveillance: MRI every 3 to 6 months, serial Ki-67/proliferation indexing, and multidisciplinary review to detect recurrence or invasion early.
